# Single-cell genomics of a bloom-forming phytoplankton species reveals population genetic structure across continents

**DOI:** 10.1093/ismejo/wrae045

**Published:** 2024-03-15

**Authors:** Raphael Gollnisch, Dag Ahrén, Karin Rengefors

**Affiliations:** Department of Biology, Aquatic Ecology, Lund University, 22362 Lund, Sweden; Department of Earth Sciences, University of Oxford, Oxford OX1 3AN, UK; National Bioinformatics Infrastructure Sweden (NBIS), SciLifeLab, Department of Biology, Lund University, 22362 Lund, Sweden; Department of Biology, Aquatic Ecology, Lund University, 22362 Lund, Sweden

**Keywords:** restriction-site associated DNA (RAD) sequencing, single-cell whole-genome amplification (WGA), Gonyostomum semen, dispersal, adaptation, ecological divergence

## Abstract

The study of microbial diversity over time and space is fundamental to the understanding of their ecology and evolution. The underlying processes driving these patterns are not fully resolved but can be studied using population genetic approaches. Here we investigated the population genetic structure of *Gonyostomum semen*, a bloom-forming phytoplankton species, across two continents. The species appears to be expanding in Europe, whereas similar trends are not observed in the USA. Our aim was to investigate if populations of *Gonyostomum semen* in Europe and in the USA are genetically differentiated, if there is population genetic structure within the continents, and what the potential drivers of differentiation are. To this end, we used a novel method based on single-amplified genomes combined with Restriction-site Associated DNA sequencing that allows de novo genotyping of natural single-cell isolates without the need for culturing. We amplified over 900 single-cell genomes from 25 lake populations across Europe and the USA and identified two distinct population clusters, one in Europe and another in the USA. Low genetic diversity in European populations supports the hypothesized recent expansion of *Gonyostomum semen* on this continent. Geographic population structure within each continent was associated with differences in environmental variables that may have led to ecological divergence of population clusters. Overall, our results show that single-amplified genomes combined with Restriction-site Associated DNA sequencing can be used to analyze microalgal population structure and differentiation based on single-cell isolates from natural, uncultured samples.

## Introduction

Phytoplankton play vital roles in biogeochemical processes and ecosystem functioning on Earth. They are responsible for a major part of oxygen production, carbon sequestration, nutrient cycling, and form the base of aquatic planktonic food webs [1, 2]. To fully understand the ecology and evolution of phytoplankton, their diversity in time and space, including their biogeographic patterns, must be studied. At the beginning of the 21st century, microbes were proposed to lack biogeographic patterns because their small size and enormous population sizes suggest essentially unlimited dispersal [3]. Instead, microbes were hypothesized to be constrained by environmental filters only [4, 5]. However, later studies showed that spatial structure of microbial diversity was driven by both geographical (physical dispersal limitation) and historical effects, as well as environmental filtering [6–8]. This can in part be attributed to methodological advances in molecular genetics that have increased the resolution to differentiate more closely related populations. Several studies now provide evidence of biogeographical patterns in both marine (e.g. [9, 10]) and freshwater (e.g. [11]) phytoplankton.

To date, the processes underlying biogeographic and population genetic structure in phytoplankton are not fully resolved. Biogeographic patterns are generated by processes at the population level, including selection, mutation, genetic drift, as well as gene flow (dispersal). Consequently, population genetic analyses are used to study these processes. In lake phytoplankton, population genetic structure and genetic differentiation between populations within species indicate that gene flow is limited (e.g. [12, 13, 14]). This limitation could be because of either physical or biological barriers [15]. The latter could, for example, be because of priority effects (advantage of first colonizers that shape the population genetic structure) and/or local adaptation (when the fitness of a local population is tweaked to the local trait optimum through selection against poorly suited phenotypes). To address which processes drive population genetic structure, studies are needed that encompass a range of distances and numerous lakes. However, few studies have been able to combine large scale sampling (across continents) and enough strains to utilize population genetic methods.

The main factor hampering population genetic studies of phytoplankton (and many other microbial eukaryotes) is the enormous sampling and culturing effort needed to collect enough strains per population. Culturing is needed to obtain enough DNA for downstream analyses, but only a minor fraction of microorganisms can be grown in the laboratory [16, 17]. Even among species that can be cultured, sampling can usually not be easily combined with immediate isolation and culturing, and the survival rate is often low. To circumvent these problems, we have applied a new method, single-amplified genomes combined with RADseq (SAG-RAD) [18], that we have developed to perform population genomic analyses on single protist cells. This approach combines whole-genome amplification from single cells (single-amplified genomes, SAG) with Restriction-site Associated DNA (RAD) sequencing to produce reduced representation sequencing libraries.

The aim of this study was to investigate the large-scale population genetic structure of the limnic phytoplankton species *Gonyostomum semen* (Raphidophyceae). We used SAG-RAD because *G. semen* is difficult to grow and has a large genome (diploid genome size 2C ≈ 32 Gbp) [14], making whole-genome sequencing an unfeasible approach. Moreover, pooling enough cells from a natural sample to avoid genome amplification is impossible. *Gonyostomum semen* is a species that forms nuisance algal blooms in freshwater sites around the world [19, 20]. An increasing incidence of blooms has been observed in northern Europe during the past five decades [19–22]. It is currently considered invasive with potential southwards range expansion in Europe, colonizing new habitats and forming nuisance blooms in a variety of freshwater systems [19, 20, 23–25]. In contrast to Europe, blooms of *G. semen* appear to be less common across North America [26].

Earlier population genetic studies of *G. semen* using monoclonal cultures and Amplified Fragment Length Polymorphism demonstrated that the lake populations in Finland and Scandinavia are genetically distinct, yet very similar [27, 28] suggesting a recent rapid expansion. Single-nucleotide polymorphisms (SNPs) obtained from RADseq on the same samples [14], suggested an east–west divide as well as a dispersal direction from the northeast/east toward the southwest/west. However, the large-scale biogeographic patterns and population structure across continents is not known, nor what shapes these patterns.

The specific aims of this study were to identify whether populations of *G. semen* in Europe and in the USA form distinct population clusters, if the genetic differentiation of populations is more pronounced between continents than within, and if there are signs of recent expansion in these populations. We further investigated whether there is a biogeographic population structure within each of the two continents, if isolation-by-distance (i.e. physical dispersal limitation) limits dispersal between populations, and if there are differences in environmental variables that are associated with the population structure.

## Materials and methods

To investigate the biogeographic pattern of *G. semen* at the population level, single cells were isolated from lakes both in Europe and in the USA to cover a wide geographic distribution. The sampled lakes were chosen to represent both the geographic distribution of *G. semen* as well as lakes of diverse physical, chemical, and biotic characteristics, reflecting the wide environmental range of *G. semen* occurrence. The genomes of single cells were analyzed using the SAG-RAD method [18].

### Sampling and single-cell isolation

**Figure 1:**
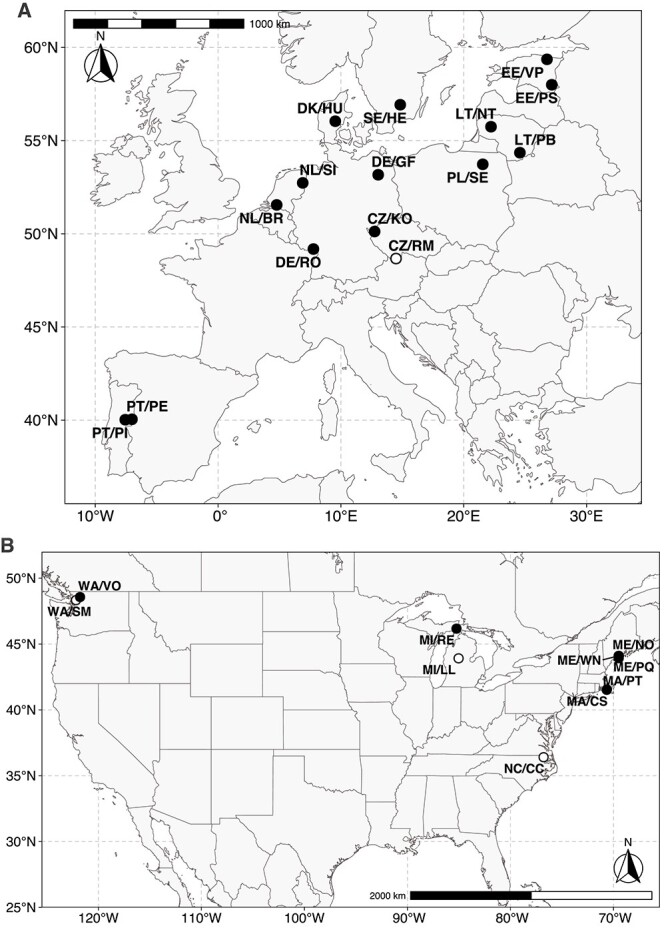
Maps of *G. semen* sampling sites in Europe (A) and in the USA (B). (A) Fifteen lakes in Europe—EE: EE/VP, EE/PS; LT: LT/NT, LT/PB; CZ: CZ/KO, CZ/RM; PL: PL/SE; DE: DE/RO, DE/GF; NL: NL/BR, NL/SI; DK: DK/HU; SE: SE/HE; PT: PT/PE, PT/PI. Population CZ/RM (white circle) was omitted in parts of the analysis because of few shared loci with other populations. See Table S1. For lake coordinates, sampling date and *G. Semen* abundance. (B) Ten lakes in the USA—WA: WA/VO, WA/SM; MI: MI/RE, MI/LL; ME: ME/NO, ME/WN, ME/PQ; MA: MA/PT, MA/CS; NC: NC/CC. Populations WA/SM, MI/CC, and NC/CC (white circles) were omitted in parts of the analysis because of few shared loci with other populations. See Table S2 for lake coordinates, sampling date and *G. semen* abundance.

*Gonyostomum semen* was sampled from lakes in Estonia, Lithuania, Czech Republic, Poland, Germany, the Netherlands, Denmark, Sweden, and Portugal in the summer of 2017 (Fig. 1A and Table S1) and in the USA in 2015 (North Carolina) and in 2018 in Washington, Michigan, Maine, and Massachusetts (Fig. 1B and Table S1). Plankton samples were collected using a plankton net (mesh size 20 μm) and filtered through a 150-μm mesh to exclude larger grazers. Single cells of *G. semen* were then isolated (32–82 per sampling site, Table S2), washed, and sorted manually using custom micropipettes as previously described [18]. Single-cell isolates were frozen immediately, transported at −20°C, and stored at −80°C until amplification.

### Multiple displacement amplification of single-cell genomes (SAGs)

Single-cell genomes of *G. semen* cells were amplified (25–58 per sampling site, Table S2) through Multiple Displacement Amplification (MDA) using the REPLI-g Single-Cell Kit (QIAGEN, Hilden, Germany) as previously described [18] to produce SAGs. Each reaction contained 0.5-μM SYTO13 (Invitrogen) fluorescent nucleic acid stain to monitor amplification curves on a real-time PCR system (CFX96 Touch, BIO-RAD). The amplification critical point (Cp) was determined from amplification curves as the *x*-value of the inflection point (i.e. the time required until the inflection point of the exponential phase is reached in an amplification). Successful MDA reactions (16–45 per sampling site, Table S2) were purified using AMPure XP beads (Beckman Coulter, Brea, CA, USA) according to the QIAGEN Supplementary Protocol RG34 replacing TE with EB buffer (QIAGEN). Fluorometric DNA quantification was performed following instructions in the QIAGEN REPLI-g Single-Cell Kit handbook. To confirm species identity, one SAG per population was amplified in two PCR assays; a *Gonyostomum*-specific 18S assay (primers GS2-F and GS5-R) [29] and a universal *cox1* assay (primers LCO1490 and HCO2198) [30]. The PCR products were sequenced using Sanger sequencing (in house) and the species identity was confirmed using BLASTN in BLAST+ v2.14.1 [31] against the NCBI nucleotide database (Table S3).

### Preparation of RADseq libraries and sequencing

Single-digest RADseq library preparation was carried out as previously described [18]. The P1 adapters used here (Table S4) contained unique 8-bp barcodes. Samples with different P1 adapters were randomly pooled into 18 libraries, each containing 34–40 samples, prior to DNA shearing.

Size selection of the sheared libraries was performed using SPRIselect beads (Beckman Coulter). A 0.75 ratio of bead suspension to sample followed by a 0.55 ratio was used to select for fragment sizes of 300–600 bp. P2 adapters (Table S4) were then ligated to each of the 18 libraries of randomly pooled samples. Each P2 adapter ligation reaction was incubated at room temperature for 60 min, followed by reaction cleanup and removal of P2 adapter dimers (left-side size selection as described above for removal of P1 adapter dimers) and eluted in 45-μl EB buffer. Following a final reaction cleanup (AMPure XP beads) after PCR amplification, the pools were sequenced (paired-end, 150 bp) on a Nova Seq 6000 S4 flow cell (Illumina) at the SNP&SEQ Technology Platform of the SciLifeLab facility in Uppsala, Sweden.

### Data processing and analysis

Sequences were processed using the software Stacks 2 v2.59 [32]. Stacks process_radtags was used for demultiplexing and to filter reads. Reads that contained adapter sequences, reads with an uncalled base, reads with low quality scores, and reads with no intact restriction enzyme cut site were discarded. This was followed by removal of potential contaminant reads using the taxonomic sequence classifier Kraken 2 v2.1.2 [33]. Additional quality assessment of all sample sequences was performed using FastQC v0.11.9 and MultiQC v1.11 [34]. Loci (RAD loci, i.e. genomic regions up- or downstream of a restriction site) were built and analyzed de novo by running the Stacks pipeline manually.

Parameters in Stacks ustacks were set to a minimum stack depth of 5 (parameter *m*) and a distance allowed between stacks of 3 (parameter *M*) based on Rengefors *et al*. [14] to maximize the number of utilized reads and polymorphic SNPs while maintaining a mean coverage of at least 20x. Loci with extreme coverage (more than three standard deviations above the mean) were excluded from further analysis. The percentage of repetitive reads (Fig. S2) was determined as the fraction of reads attributed to those loci that were excluded because of extreme coverage. To mitigate effects of missing data caused by individuals with a high amount of missing loci and to ensure retrieval of sufficient loci across samples [35], samples with <50 000 putative loci were excluded from further analysis.

To create the catalog of consensus loci, representative samples with the highest mean coverage within the upper 0.5 quantile of putative loci were selected, restricting the maximum number of individuals per lake to half of its total number of individuals (resulting in five to eight samples per lake and 187 samples in total). The catalog was created in eight steps using cstacks, adding one sample per lake in each step (Fig. S4) and allowing for three mismatches between sample loci (parameter *n*).

Informative loci were determined in Stacks populations by specifying the minimum number of populations a locus must be present in (*p*) and the minimum proportion of individuals in a population a locus must be present in (*r*). Only the first SNP per RAD locus was utilized for population genetic analyses to avoid using multiple linked neighboring SNPs. Out of 657 sequenced samples in the RAD library, 422 were retained, corresponding to 5–34 samples per sampling location (Table S2). Stacks populations runs were carried out on three data sets with the following: *p* = 13 and *r* = 0.7 for all 15 lakes in Europe, *p* = 7 and *r* = 0.7 for a data set of seven lakes in the USA, and *p* = 20 and *r* = 0.6 when combining 22 lakes from both continents in one analysis. Lakes Washington (WA): Summer Lake (WA/SM), Michigan (MI): Lost Lake Fen (MI/LL), and North Carolina (NC): Colly Creek (NC/CC) in the USA were excluded from both data sets because of an extremely low number of loci (< 2%) shared among populations. The software R v3.6.3 [36] with dplyr v1.1.2 [37] was used in several analysis steps and the packages ggplot2 v3.3.0 [37] and ggpubr v0.2.1 were used for plotting. Clone correction analysis using poppr v2.9.4 [38] identified no duplicated genotypes. Maps of sampling sites were plotted using the additional R packages rnaturalearth v0.1.0, ggspatial v1.0.3 and sf v0.9-6 [39]. The Cp (i.e. time at the inflection point of the amplification curve) of MDA reactions was determined from amplification curves using qpcR v1.4-1 [40].

### Population genetic analyses

Population differentiation (*F*_ST_), and nucleotide diversity (π), observed and expected heterozygosity (*H*_o_, *H*_e_) were calculated directly in Stacks based on all SNPs utilized by the Stacks populations (i.e. the first SNP of each RAD locus). Calculations of nucleotide diversity were based on all called SNPs including low frequency variants. Lake Czechia (CZ): Rychnov nad Malší (CZ/RM) in Europe was excluded in subsequent analyses of coancestry (fineRADstructure), discriminant analysis of principal components (DAPC), and significant isolation by distance (IBD), because of a high number of missing loci. The population structure of the *G. semen* populations in Europe and in the USA was analyzed using several independent methods. A clustered coancestry matrix of all populations from Europe and the USA combined was created in fineRADstructure [41], using a missing data cutoff of 40%. For MCMC in fineRADstructure, 500 000 burn-in iterations were followed by 500 000 sample iterations, and a thin interval of 5000. The coancestry matrix contains counts the number of most similar haplotypes (RAD loci) in pairwise comparisons of individuals. Population structure was further investigated using the Bayesian assignment approach implemented in Structure v2.3 [42, 43]. The analysis was performed separately for each of the two continents with the clue for K ranging from 1 to 8 (USA) or from 1 to 15 (Europe), assuming an admixture model, correlated allele frequencies, and without location priors. A burn-in period of 50 000 steps followed by 100 000 additional repetitions were performed in each Structure run. For each K, 20 independent iterations were performed. The most likely K to describe population clusters in the data was estimated with the Evanno method [44, 45] using the R package pophelper v2.3.1. Structure plots were visualized using CLUMPAK [46]. Analysis of molecular variance (AMOVA) was performed in GenoDive v3.05 [47]. DAPC was performed on the combined data set, as well as on the Europe and the US data sets separately in R with the packages adegenet v2.1.5 [48] and vegan v2.5-7. The optimal number of principle components (PCs) to retain in DAPC was determined thorough cross-validation and a-score optimization. Previously published environmental variables of the different lakes [49] were superimposed on the DAPC ordination plots. No environmental data was available for lakes CZ: Komáří Rybnik (CZ/KO) and Sweden (SE): Helgasjön (SE/HE). To test significant IBD, a Mantel test of genetic distances against geographic distances was performed in R with package ade4 v1.7-18 [50]. This analysis was performed separately for populations in the USA, in Europe, and for the eastern and western population clusters in Europe as identified by the Structure analysis.

## Results

### Library demultiplexing, read filtering, and de novo assembly of RAD loci

The total number of reads in all 18 sequenced RAD libraries of randomly pooled SAGs was 15.3 billion, 98.3% of which were retained after demultiplexing and quality filtering through Stacks process_radtags. Around 5.6% of all reads were identified as potential contaminant reads through taxonomic sequence classification using Kraken (Fig. S1) and were discarded. Evaluation of amplification curves revealed a strong positive correlation of the amplification Cp and the percentage of repetitive reads found in a sample, along with a strong negative correlation of Cp and the number of putative loci that are recovered (Fig. S2). This suggests that exclusion of sequenced SAG-RAD samples during the analysis because of missing data could be minimized by evaluating MDA amplification curves and selection of SAGs for sequencing based on the quality of the amplification. Genome recovery (i.e. the number of loci obtained from a sample) was strongly correlated with the timing of amplifications throughout all populations in this study.

After exclusion of samples for which <50 000 loci were recovered, the mean number of putative loci was 92 995 (mean coverage 77.8×) per sample (see Fig. S3 for all samples before filtering). The final catalog created using Stacks cstacks from 187 samples contained a total of 2 498 256 loci (Fig. S4). Across the 15 populations from lakes in Europe, 664 variant sites were identified, that were present in 70% of the individuals or more in at least 13 of the populations (Table 1). Across the seven populations from lakes in the USA, 708 variant sites were identified that were shared among at least 70% of the individuals in all seven populations (Table 1). In the combined data set with all 22 lake populations from both continents, 2452 variant sites were identified in at least 60% of the individuals in 22 populations or more. To keep the amount of missing data in the analysis to a minimum, most of the analyses of population differentiation and genetic structure were performed on separate data sets of populations in Europe and populations in the USA.

**Table 1:** Population genetic metrics of *G. semen* lake populations in Europe and in the USA. Table contains population ID, number of individuals in each population, number of all sites (variant and fixed), percentage of polymorphic sites, number of private alleles, number of variant sites, nucleotide diversity π, observed heterozygosity *H*_o_, expected heterozygosity *H*_e_, and the inbreeding coefficient *F*_is_. Europe: analysis based on a total number of 664 variant sites from 236 individuals. USA: analysis based on a total number of 708 variant sites from 124 individuals.

	**Pop.**	**Indiv.**	**Sites**	**% Polym.** **sites**	**Private** **alleles**	**Variant** **sites**	***π***	***H***_**o**_	***H***_**e**_	***F***_**is**_
Europe	CZ/KO	14	713 473	0.010	15	634	0.024	0.024	0.023	0.002
	CZ/RM	11	283 227	0.032	53	250	0.119	0.115	0.112	0.014
	DE/GF	11	718 891	0.005	6	639	0.012	0.014	0.011	−0.005
	DE/RO	21	713 959	0.012	20	645	0.020	0.019	0.019	0.003
	DK/HU	15	696 211	0.010	14	619	0.018	0.016	0.018	0.011
	EE/PS	7	722 635	0.006	7	650	0.018	0.018	0.017	0.002
	EE/VP	9	683 719	0.007	6	609	0.020	0.016	0.018	0.010
	LT/NT	16	705 947	0.007	13	630	0.012	0.011	0.012	0.006
	LT/PB	25	670 825	0.035	111	611	0.037	0.027	0.036	0.070
	NL/BR	34	729 975	0.023	35	656	0.028	0.024	0.027	0.020
	NL/SI	23	730 964	0.024	39	656	0.034	0.028	0.033	0.028
	PL/SE	13	579 642	0.011	21	515	0.020	0.018	0.019	0.003
	PT/PE	14	706 333	0.008	11	636	0.015	0.016	0.014	−0.003
	PT/PI	15	660 157	0.008	17	601	0.015	0.016	0.015	0.001
	SE/HE	8	645 303	0.008	11	583	0.020	0.014	0.018	0.012
USA	MA/CS	20	691 287	0.022	52	708	0.039	0.025	0.038	0.049
	MA/PT	20	691 275	0.021	50	708	0.046	0.031	0.044	0.044
	ME/NO	5	691 148	0.014	58	708	0.044	0.032	0.039	0.031
	ME/PQ	15	691 229	0.027	83	708	0.050	0.032	0.048	0.062
	ME/WN	13	691 174	0.022	54	708	0.044	0.030	0.042	0.044
	MI/RE	23	691 228	0.023	68	708	0.046	0.047	0.045	0.005
	WA/VO	28	691 282	0.030	108	708	0.057	0.061	0.056	−0.005

### Population genetic metrics

The analysis of population genetic metrics (Table 1) was based on a total number of 664 variant sites from 236 individuals in Europe and 708 variant sites from 124 individuals in the USA. The percentage of polymorphic sites among all sites was overall very low and ranged from 0.005 (Germany (DE): Große Fuchskuhle (DE/GF)) to 0.035 (Lithuania (LT): Pabezninkai (LT/PB)) in Europe and 0.014 (Maine (ME): Nobleboro (ME/NO)) to 0.03 (WA: Vogler Lake (WA/VO)) in the USA. The mean nucleotide diversity π of variant sites ranged from 0.012 to 0.119 across all populations with values in the USA generally exceeding those in Europe (Wilcoxon signed-rank test: *p* = 0.001). In Europe, π varied mostly between 0.012 (DE/GF and LT: Natalka (LT/NT)) and 0.037 (LT/PB), but was as high as 0.119 in CZ/RM. In the USA, values for π ranged from 0.039 (Massachusetts (MA): Cedar Swamp (MA/CS)) to 0.057 (WA/VO). Compared with the other populations, CZ/RM only shared a relatively low number of 250 variant sites (283 227 variant and fixed sites) with other populations in Europe and had a relatively high number of 53 private alleles (i.e. an allele that is found in only one of several populations). The number of private alleles varied strongly between populations and ranged from 6 (Estonia (EE): Viitna Pikkjärv (EE/VP) and DE/GF) up to 111 (LT/PB). In the USA, the number of private alleles ranged from 50 (MA: Peterson Pond (MA/PT)) to 108 (WA/VO). Heterozygosity (a measure of genetic variation in a population) was overall very low, especially in European populations, where mean values of observed heterozygosity varied between 0.011 (LT/NT) and 0.028 (the Netherlands (NL): Siepeldijk (NL/SI)) for all populations except CZ/RM with 0.115. In the USA, observed heterozygosity was slightly higher and ranged from 0.025 (MA/CS) to 0.061 (WA/VO). Mean values of expected heterozygosity were similar but tended to be slightly higher than those of observed heterozygosity as is also indicated by inbreeding coefficients (*F*_is_) ranging from −0.005 (DE/GF and WA/VO) to a maximum of 0.07 (LT/PB).

A combined analysis of 2452 loci in 360 individuals from lake populations across Europe and the USA (Table S6) showed similar patterns in terms of a generally higher number of private alleles, higher nucleotide diversity, and higher heterozygosity in the USA compared with Europe.

### Population differentiation

**Figure 2:**
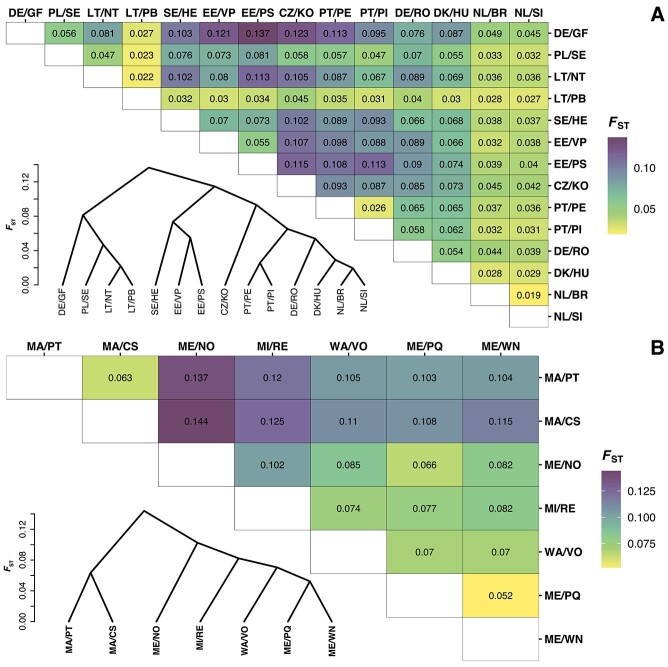
Pairwise genetic distance (*F*_ST_ values) between lake populations of *G. semen* in Europe^*^ (A) and in the USA (B). Upper right: Heatmap table of *F*_ST_ values. Lower left: hierarchical clustering tree that was used to arrange the order of population in the heatmap table. ^*^F_ST_ values between population CZ/RM and all other populations ranging from 0.309 to 0.447 were omitted from the heatmap table.

Analysis of the SNP data from *G. semen* lake populations in Europe and in the USA showed genetic differentiation between all lake populations. Overall, values of pairwise genetic distance (*F*_ST_) between lake populations were significant and ranged from moderate to high differentiation. In Europe (Fig. 2A), lowest *F*_ST_ values around 0.019–0.026 were generally found between lakes that were in relative proximity. Population CZ/RM appeared highly differentiated from all other lakes in Europe and very high *F*_ST_ values, ranging from 0.309 to 0.447, were observed between CZ/RM and all other lakes. *F*_ST_ values were generally higher in the USA (Fig. 2B), even between populations within a few kilometers of distance, and ranged from 0.052 (ME: West Neck Pond (ME/WN) and Pemaquid River (ME/PQ)) up to around 0.14 for pairwise comparisons between the Massachusetts lakes and ME/NO. With all *F*_ST_ values above 0.1, the two lakes in Massachusetts appeared to be highly differentiated from all other lakes in the USA.

In a combined analysis of lakes across Europe and the USA, *F*_ST_ values between lakes within each of the two continents were generally lower than between lakes on different continents (Fig. S5). The lowest *F*_ST_ values were observed between lakes within Europe and values between lakes within the USA were overall higher. However, the ranges of *F*_ST_ values between and within continents were not clearly distinct and largely overlapped.

### Population genetic structure between continents

Population structure of the *G. semen* populations in Europe and in the USA was analyzed using several independent methods. Clustering of populations generally correlated with geography, both across and within Europe and the USA.

A combined DAPC of populations in Europe and in the USA revealed a distinct separation of European and US population clusters (Fig. S6). An AMOVA of *G. semen* populations across Europe and the USA showed significant differentiation of populations both within (35.3% of the variation; *F* = 0.38; *p* = 0.001) and between continents (37.9% of the variation; *F* = 0.57; *p* = 0.001).

**Figure 3:**
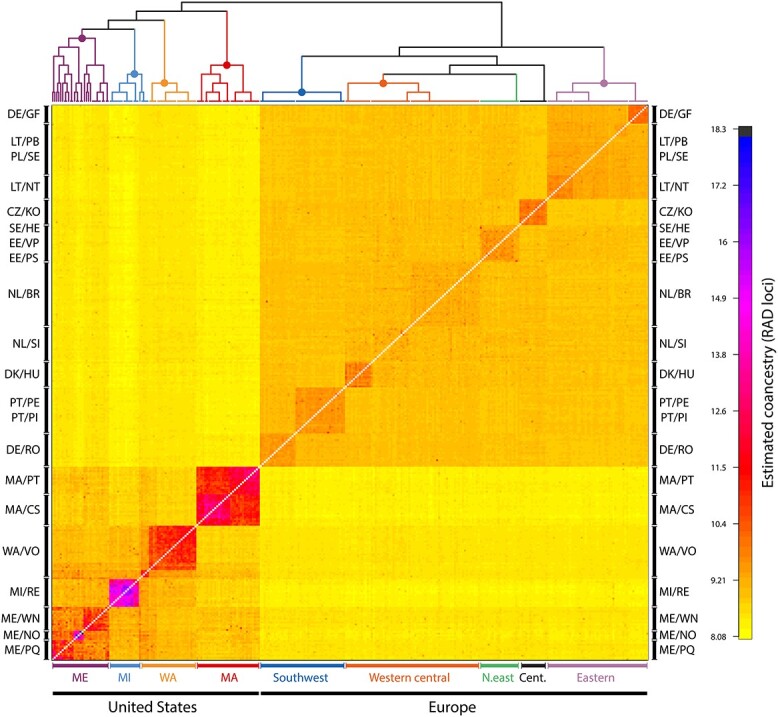
Clustered fineRADstructure coancestry matrix of *G. semen* populations in Europe and in the USA. The heat map depicts pairwise coancestry (i.e. the number of most similar haplotypes (RAD loci) in pairwise comparisons of individuals) based on RAD loci among individuals according to the color scale shown on the right. Labels on the left and right denote clusters of individuals in lake populations. Bottom labels denote major geographic clusters of populations on the two different continents as indicated by the hierarchical clustering tree (color coded) on top of the coancestry matrix.

Analysis of coancestry (nearest neighbor haplotype relationships) between all populations in the USA and in Europe using fineRADstructure (Fig. 3) also showed that European and US populations form distinct clusters. Estimated coancestry within population clusters was generally higher in the USA than in Europe. Ten clusters on five main branches were identified within the European population cluster. These included an eastern branch with the populations LT/NT, LT/PB, Poland (PL): Sęczek (PL/SE), and DE/GF. The population CZ/KO formed a separate central branch. A western central branch consisted of Denmark (DK): Hundsø (DK/HU), NL/SI, and NL: Breda Zuid Oost (NL/BR). A northeast branch included the populations SE/HE, EE/VP, and EE: Partsi Saarjärv (EE/PS). And a southwest branch contained the populations DE: Rohrwoog (DE/RO), Portugal (PT): Penha Garcia (PT/PE), and PT: Pisco (PT/PI). In the USA, the coancestry analysis identified seven clusters on four main branches, separated by states.

### Population genetic structure within continents

**Figure 4:**
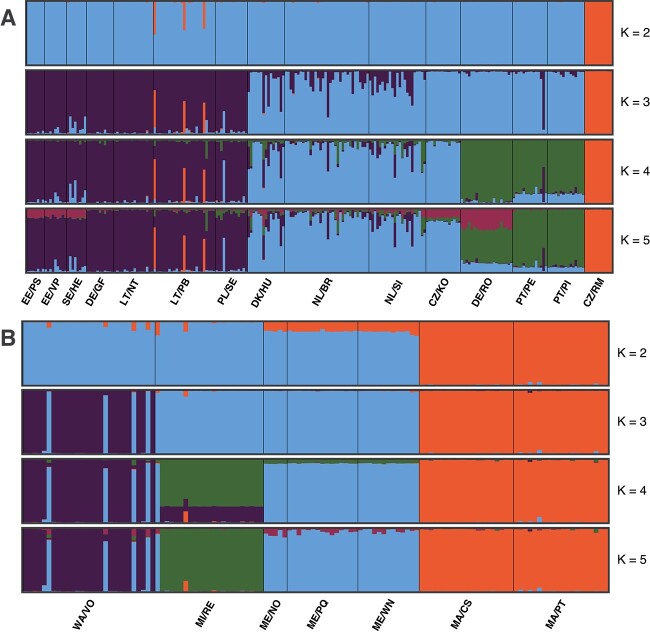
Structure plots of *G. semen* lake populations in Europe (A) and in the USA (B) for clustering values *K* 2–5. Each individual strain is represented by a vertical bar, which is partitioned into *K*-colored segments that represent estimated assignment fractions of strains in *K* population clusters (along the *y-*axis). Bottom labels indicate country/abbreviation of lake names (see Fig. 1).

Fine scale population structure was analyzed separately for populations in Europe and in the and USA using the program Structure. Based on the Evanno method [45], the best clustering of populations in Europe was with two inferred population clusters (*K* = 2, Fig. S7), separating population CZ/RM from all other populations (Fig. 4A). However, more clusters were identified and the log-likelihood for higher values of *K* increased until *K* = 5, showing distinct western and eastern clusters with *K* = 3. For *K* = 4, the western cluster was further divided into a central European cluster and the three populations DE/RO, PT/PE, and PT/PI in the southwest formed a separate cluster. By including a fifth group (*K* = 5) no additional cluster was formed. In the Structure analysis of populations in the USA (Fig. 4B), the best clustering was with three inferred population clusters (*K* = 3, Fig. S8). These included one cluster of both lakes in Massachusetts, a second cluster formed by the lakes in Maine, with Michigan and Washington as a third cluster. The Michigan lake population was separated from the Maine populations for higher values of *K*.

### Potential drivers of population differentiation

**Figure 5:**
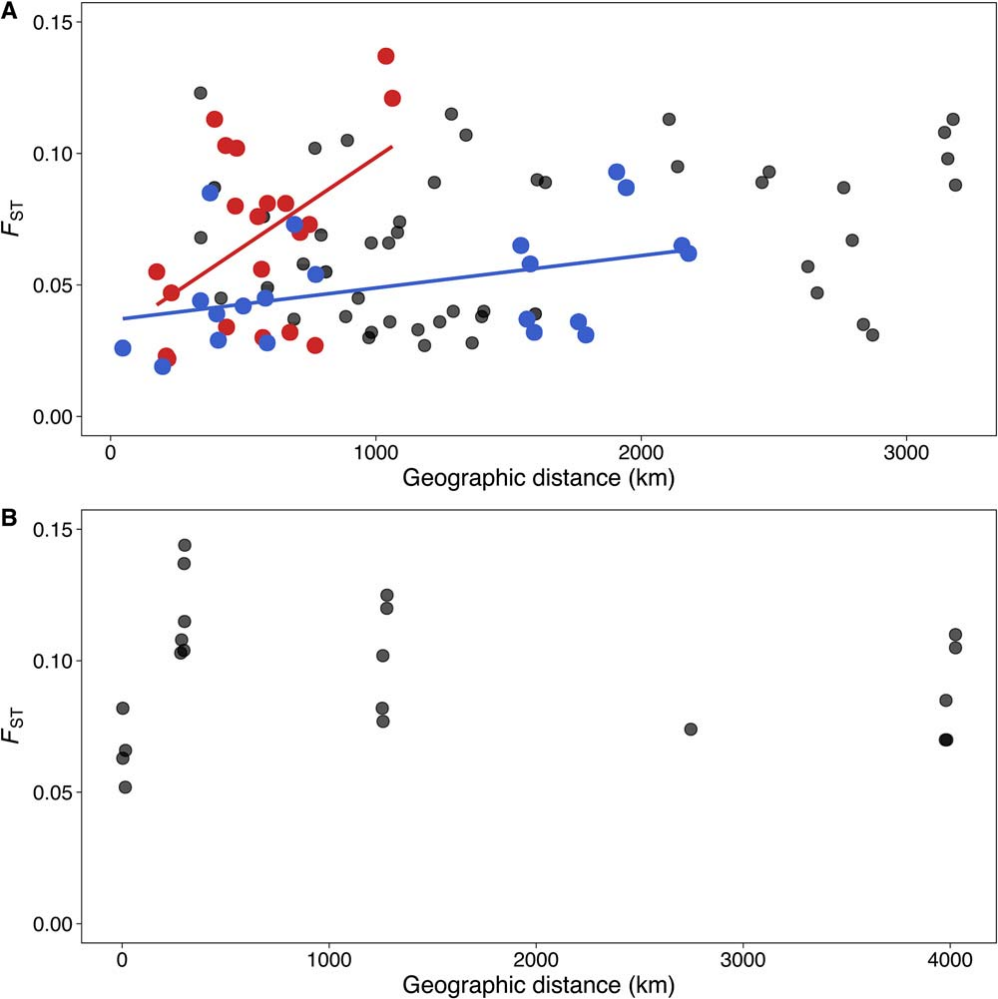
IBD of *G. semen* populations in Europe (A) and in the USA (B). Genetic distance (*F*_ST_) is plotted against geographic distance. (A) Europe—no IBD overall (black), IBD significant in the eastern cluster (red with linear fit; *R*^2^ = 0.24), IBD significant in the western cluster (blue with linear fit; *R*^2^ = 0.17); (B) USA—no IBD overall.

Overall, population differentiation was correlated with geographic distance between lake populations within a certain range of geographic distance, but not across the full geographic scale. IBD across all populations in Europe (Fig. 5A) was not significant (Mantel statistic *r* = 0.22, *p* = 0.118). However, when population genetic clusters according to Structure were used, another pattern emerged. The eastern cluster (containing lake populations in Estonia, Sweden, Lithuania, and northeastern Germany) showed significant IBD (Mantel statistic *r* = 0.49, *p* = 0.028). Significant IBD (Mantel statistic *r* = 0.41, *p* = 0.038) was also found in the western cluster (containing lake populations in Denmark, the Netherlands, southwestern Germany, and Portugal). In contrast, the relationship between geographic distance and genetic differentiation was slightly negative in the USA (Fig. 5B) and there was no sign of IBD between populations (Mantel statistic *r* = −0.14, *p* = 0.569).

**Figure 6:**
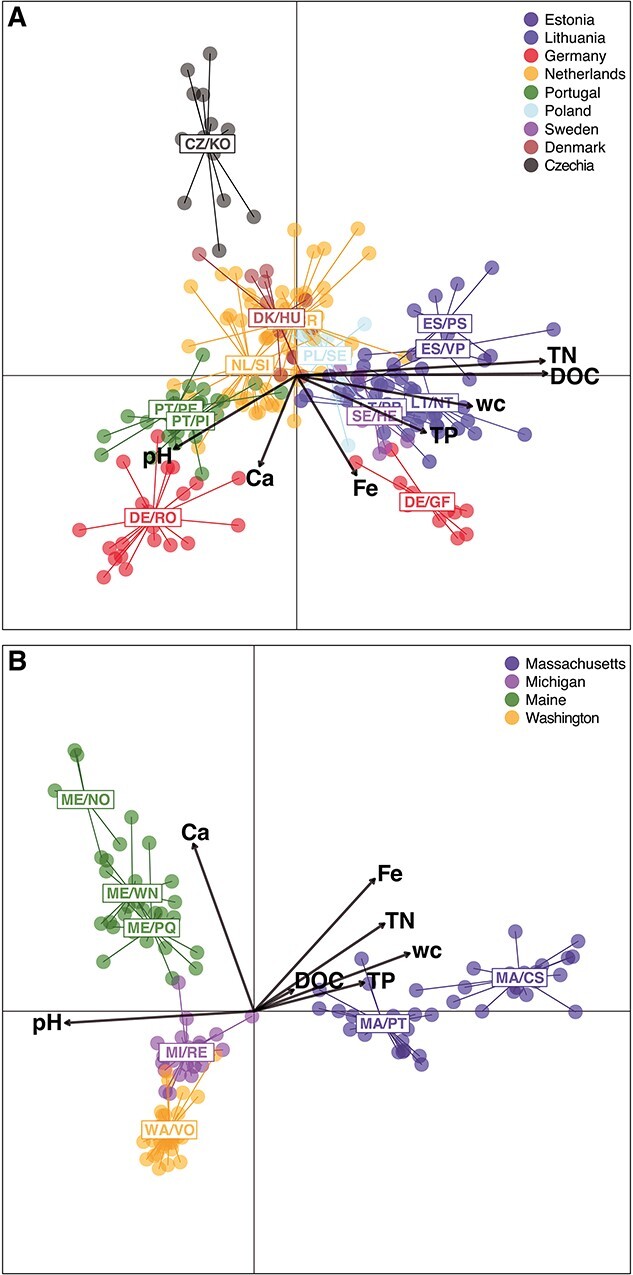
DAPC of *G. semen* lake populations within continents. Points represent individuals and lines connect individuals within populations. The number of retained PCs was determined using cross-validation and a-score optimization. Color code denotes country or state (USA). (A) In Europe, retaining 13 PCs. (B) In the USA, retaining 12 PCs. Vectors of environmental variables are superimposed on the DAPC ordination scores. Superimposed environmental variables (from [49]) are pH, wc, and concentrations of Ca, Fe, TP, TN, and DOC. No environmental data is available for lakes CZ/KO and SE/HE.

Separate DAPC analyses of Europe (Fig. 6A) and the USA (Fig. 6B) generally showed a clustering pattern that reflected their geography. In Europe, a northeastern, a central and a southwestern cluster were observed. Population CZ/KO in Czech Republic formed a separate cluster. The two populations in Germany were separated with DE/RO being part of the southwestern and DE/GF being part of the northeastern cluster. In the USA, three separate population clusters were identified, similar to the Structure analysis. The populations in Maine and Massachusetts formed two separate clusters adjacent to a cluster including WA/VO and MI: Rexton Bog (MI/RE).

Environmental vectors plotted on the DAPC ordination showed that the separation of the western cluster in Europe correlated with higher pH and calcium (Ca) concentration. The eastern cluster, in contrast, correlated with higher concentrations of iron (Fe), total phosphorus (TP), total nitrogen (TN), dissolved organic carbon (DOC), and higher water color (wc). In the USA, separation of the two lake populations in Massachusetts was mainly correlated with lower pH and higher concentrations of Fe, TN, TP, as well as higher wc, compared with the other lakes. The separation of the three lake populations in Maine was correlated with higher concentrations of Ca.

## Discussion

Here we present a phytoplankton population genomic study based on single cells isolated directly from lake samples without culturing. We accomplished this by using the new method SAG-RAD, which is based on SAGs and reduced representation sequencing, followed by SNP analyses. Our main finding was that *G. semen* forms two distinct geographic clusters, one in Europe and one in the USA, demonstrating a substantial dispersal barrier between the continents. In addition, we detected geographic population structure of *G. semen* within each of the two continents connected to environmental factors and isolation-by-distance. Below we discuss these results in relation to dispersal and distribution of phytoplankton, as well as considerations when using SAG-RAD.

Although *G. semen* is widely distributed, it forms two distinct genetic clusters that separate the populations in Europe and in the USA. This was clear both in the DAPC and fineRADstructure coancestry analyses, which are analyses that are based on very different assumptions, where DAPC does not rely on a particular population genetic model [51]. This distinct pattern implies that gene flow between the continents is unlikely and that the dispersal barriers are high. Given that the two populations are separated by an ocean, this finding was not unexpected, but demonstrates that although *G. semen* is a cosmopolitan species, the populations are highly differentiated.

Within both Europe and the USA, we observed clear geographic clustering based on coancestry (genetic similarity) analyses. There were also differences in intra-population coancestry, with a more pronounced isolation of populations in the USA compared with Europe. Possibly, this difference is related to the direction of the isolation. Within the USA, most of the isolation among lakes is on an east–west scale, which means a greater disconnection in terms of dispersal vectors such as birds, whose flyways tend to be in a north–south direction [52]. Pairwise genetic differentiation (*F*_ST_) was generally higher in lake populations between Europe and the USA, compared with *F*_ST_ of populations within each continent. However, this was not always the case, despite that the USA and Europe are separated by around 5000 km across the Atlantic Ocean. This suggests the relevance of other factors than physical isolation through geographic distance driving differentiation of *G. semen* populations, such as environmental factors leading to local adaptation [53].

In Europe, we found a split between a northeastern and a southwestern cluster of lake populations. The Structure analysis indicated an eastern cluster of lakes in Estonia, Lithuania, Sweden, Poland, and north-east Germany, that was separated from a western cluster spanning lakes in Denmark, the Netherlands, Czech Republic, south-west Germany and Portugal. A divide into an eastern and western population cluster, along a similar longitudinal axis, was observed in Northern Europe between Sweden and Finland in the east and Norway in the west [14]. Drivers behind this separation remain unclear but could potentially be connected to bird migratory paths [14, 54]. In the USA, we similarly observed a clear separation of three distinct population clusters that coincided with the geography of the sampled lakes. These clusters were separated by latitude, further strengthening the bird migratory pathway hypothesis. Coleman [55] found a similar pattern in the green alga *Pandorina morum* indicating that the east coast, mid-west, and west coast did not share lineages. Both Coleman’s and our study suggest that east/west transport by westerlies (wind) was less effective than north–south transport (by migrating waterfowl), because their flyways correspond to these divisions [56]. Although bird transport of *G. semen* has not been studied, transport likely occurs through resting cysts, which are more tolerant than motile cells and easily germinate in non-local water [57]. Moreover, Tesson and Santl-Temkiv [58] showed that *G. semen* cells and cysts did not tolerate freezing and desiccation, indicating poor airborne dispersal.

Three populations in the USA (WA/SM, MI/LL, and NC/CC) and to a lesser degree CZ/RM in Europe appeared to be highly differentiated from other populations and had to be omitted in the analysis, or parts thereof. Both microscopic inspection and the partial 18S rRNA gene sequences clearly suggested *G. semen* for all isolates. However, although the partial sequence of *coxI* matched *G. semen* in both NC/CC and MI/LL albeit with a lower percent identity, WA/SM had a weak match to *Chattonella marina var. antiqua.* Because it is unlikely that the lineage belongs to *C. marina* (reported from brackish but not freshwater habitats) and the WA/SM lineage had high similarity to *G. semen* in the 18S rRNA gene, the species identification remains unclear. This raises the question whether these lineages represent cryptic species.

Overall, we observed higher nucleotide diversity, higher heterozygosity, and more polymorphic loci in lake populations of *G. semen* in the USA compared with populations in Europe. This suggests that populations in the USA could have been separated for a longer time than those in Europe, supporting our hypothesis of a more recent expansion of *G. semen* in Europe. Although we cannot directly infer directionality from our data, a possible scenario underlying the observed differences in genetic diversity between populations on the two continents is that the European populations of *G. semen* represent a lineage that diverged from an ancestral lineage in the USA followed by a recent expansion in Europe.

Physical dispersal limitation, or geographic distance, does play a role in generating the population genetic patterns we observed in *G. semen*, but not at all distance ranges or in all directions. Lakes in close geographic proximity usually had a low but significant genetic distance (*F*_ST_ value) both in the USA and in Europe. We further observed a trend of higher genetic distance with higher geographic distance both within the eastern and the western cluster in Europe, but not when combined. We conclude that the correlation between geographic distance and genetic differentiation seems to break down at larger distances. In the USA, we found no signs of isolation-by-distance across the long distances between populations included in this study, which as indicated above could be because of main dispersal by migratory birds. Although water, wind, and animal vectors [59, 60] are potential means of microalgal dispersal, the scales, and the extent at which these drivers act are largely unknown. Sassenhagen *et al*. [28] suggested the relevance of different dispersal mechanisms at different geographic scales and emphasized the potential role of animal vectors for dispersal between adjacent lakes. At larger geographic scales, patterns of population differentiation might be influenced by different source populations, migratory birds, and more stochastic dispersal events [28]. Finally, priority effects of first colonizers or genetic anchoring effects could explain limited gene flow between populations independent of dispersal vectors or geographic isolation [14].

Environmental selection likely also played a role in population differentiation of *G. semen*. We observed differences in environmental variables between population clusters within Europe and the USA. These environmental differences were mainly high pH and high Ca concentration on the one hand, and higher wc and high concentrations of TN, TP, DOC, and Fe on the other hand. Among the latter are conditions that are typically associated with a dominance of *G. semen* [61] and represent the characteristics of waterbodies where high density blooms of the species were first described (e.g. [62]). In the laboratory, we have shown that *G. semen* growth requires high Fe [63] and field observations connect Fe and presence of *G. semen* blooms [64]. Moreover, in Gollnisch *et al*. [49] we showed that *G. semen* distribution may be limited by a combination of high pH and high Ca concentration. The observed environmental heterogeneity could cause local adaptation within population clusters [65], limiting gene flow between different environmental clusters and thus facilitating genetic differentiation [66].

We observed relatively low genetic diversity, i.e. expected and observed heterozygosity in this data set compared with Rengefors *et al*. [14]. This is likely because of allelic dropout (ADO) during single-cell MDA, when the amplification is biased toward one allele in the presence of two alleles in a heterozygous individual resulting in an underestimation of heterozygosity. Rates of ADO are generally higher in SAG-RAD compared with RAD from extracted DNA of clonal cultures [18]. A critical step in the analysis of SAG-RAD libraries that can cause ADO is likely the construction of the RAD loci catalog. For large data sets, the number of samples used to build the catalog in the Stacks analysis should generally be limited to around 40–200 samples to minimize noise in the catalog [67]. This requires selecting a subset of samples with high coverage that is representative of the genetic diversity in the data set [67]. Therefore, in this study we selected 187 individuals (out of 422), based on number and coverage of loci, to construct the RAD catalog. Construction of the catalog from a subset of samples can, however, cause loss of low minor allele frequency variants in the data set [67]. If an allele is not included in the catalog at this step, it would be discarded when sample loci are later mapped against the catalog and cause ADO during RAD analysis. Heterozygosity could therefore be another criterion, besides number and coverage of loci, to select representative samples used to build the catalog, especially in the analysis of SAG-RAD libraries.

In this study we demonstrated that SAG-RAD provides an important alternative to traditional culture-based studies of population genetics in microalgae by circumventing major limitations associated with algal culturing. However, when using SAG-RAD, the extent of genome recovery and allelic dropout in samples, i.e. true heterozygous sites may appear as homozygous, need to be considered. This effect is because of amplification bias in MDA [68], the method used for amplification of single-cell genomes in SAG-RAD. We show that Cp can be used as a proxy for amplification quality prior to sequencing (Fig. S2).

To conclude, single-cell population genomics using SAG-RAD enabled us to analyze the genetic structure and differentiation of *G. semen* across a large geographic distance including over 20 lakes and hundreds of individuals. This allowed us to identify two distinct population clusters separating *G. semen* in Europe and in the USA indicating a divergence of the species that predates the present distribution. We found that genetic differentiation of the populations is influenced by both physical dispersal limitation and different environmental conditions. Finally, the relatively low genetic diversity in European populations supports the hypothesized recent expansion of *G. semen*. In the future, when a genome will be available for this important bloom-forming species, we anticipate that our population genomic data can be used to further investigate genes under selection, local adaptation, and links between genotypes and phenotypes.

## Supplementary Material

gollnisch-et-al_supplement_final_wrae045

## Data Availability

Analysis code is available on GitHub at https://github.com/RGollnisch/Gsemen_SAG-RAD and on Zenodo. Sequence data are available through the NCBI SRA database (BioProject ID PRJNA988296) at https://www.ncbi.nlm.nih.gov/bioproject/PRJNA988296.
