# Supplementary material for: Single-cell genomics of a bloom-forming phytoplankton species reveals population genetic structure across continents
Source: ISME J. 2024 Mar 15;18(1):wrae045. doi: 10.1093/ismejo/wrae045 (PMC11065318; doi:10.1093/ismejo/wrae045)
Supplement: gollnisch-et-al_supplement_final_wrae045 [file gollnisch-et-al_supplement_final_wrae045.pdf]

SUPPORTING INFORMATION for

Single-cell genomics of a bloom-forming phytoplankton species reveals population genetic structure across continents

Raphael Gollnisch[1,2]*, Dag Ahrén[3], Karin Rengefors[1]

[1] Lund University, Department of Biology, Aquatic Ecology; Lund, Sweden

[2] University of Oxford, Department of Earth Sciences, Oxford, United Kingdom

[3] National Bioinformatics Infrastructure Sweden (NBIS), SciLifeLab, Department of Biology, Lund University, Sweden

* Corresponding author: Raphael Gollnisch, raphael.gollnisch@gmail.com

University of Oxford, Department of Earth Sciences, South Parks Road, Oxford OX1 3AN, UK

Running title: Protist single-cell population genomics

Keywords: Restriction-site Associated DNA (RAD) sequencing, single-cell Whole Genome Amplification (WGA), *Gonyostomum semen*, dispersal, adaptation, ecological divergence

Table S1: Coordinates of *G. semen sampling* sites in Europe and in the United States with sampling date and *G. semen* abundance at the time of sampling. See Gollnisch et al. [1] for further details. # Southwest basin of artificially divided lake [2]; § from Gollnisch et al. [1]; * Southernmost side of Damariscotta Lake.

	Country/State	Sampling site	Coordinates	Sampling date	*G. semen* counts (cells L^{-1}) §
EUROPE	ESTONIA	Viitna Pikkjärv (VP)	59.443, 26.011	2017-08-03	18 400
		Partsi Saarjärv (PS)	57.997, 27.165	2017-08-01	119 570
	LITHUANIA	Natalka (NT)	55.744, 22.218	2017-08-07	494 140
		Pabezninkai (PB)	54.351, 24.570	2017-08-10	197 400
	CZECH REPUBLIC	Komáří Rybnik (KO)	50.121, 12.743	2017-08-21	NA
		Rychnov nad Malší (RM)	48.671, 14.485	2017-08-22	41 000
	POLAND	Sęczek (SE)	53.728, 21.547	2017-08-12	12 130
	GERMANY	Rohrwoog (RO)	49.181, 7.761	2017-08-17	64 600
		Große Fuchskuhle (GF) #	53.167, 13.033	2017-09-18	782 000
	NETHERLANDS	Breda Zuid Oost (BR)	51.549, 4.771	2017-08-25	229 000
		Siepeldijk (SI)	52.732, 6.890	2017-08-26	48 900
	DENMARK	Hundsø (HU)	56.040, 9.541	2017-09-14	4 850
	SWEDEN	Helgasjön (HE)	56.924, 14.830	2017-09-08	NA
	PORTUGAL	Penha Garcia (PE)	40.047, -7.015	2017-11-21	11 500
		Pisco (PI)	40.02, -7.558	2017-11-21	76 800
UNITED STATES	WASHINGTON	Vogler Lake (VO)	48.571, -121.773	2018-06-25	400
		Summer Lake (SM)	48.333, -122.168	2018-06-27	600
	MICHIGAN	Rexton Bog (RE)	46.170, -85.238	2018-07-04	2100
		Lost Lake Fen (LL)	43.915, -85.034	2018-07-06	500
	MAINE	Nobleboro (NO) *	44.104, -69.474	2018-07-11	10 300
		West Neck Pond (WN)	44.102, -69.506	2018-07-12	68 700
		Pemaquid River (PQ)	43.964, -69.510	2018-07-12	3 830
	MASSACHUSETTS	Peterson Pond (PT)	41.553, -70.640	2018-07-09	196 000
		Cedar Swamp (CS)	41.525, -70.657	2018-07-13	1 186 000
	NORTH CAROLINA	Colly Creek (CC)	34.652, -78.464	2015-07-16	NA

Table S2: Number of single cells (SC) isolated, single cell amplified genomes (SAGs) produced, SAGs sequenced, and samples with more than 50,000 putative loci (stacks) from sampling sites in Europe and in the United States.

	Site	Single cells isolated	SC genomes amplified	SAGs sequenced	>50,000 loci
EUROPE	EE/VP	40	35	17	9
	EE/PS	34	32	17	7
	LT/NT	36	36	17	16
	LT/PB	73	54	32	25
	CZ/KO	74	54	30	14
	CZ/RM	73	40	26	11
	PL/SE	56	36	27	13
	DE/RO	78	58	33	21
	DE/GF	60	34	23	11
	NL/BR	72	54	45	34
	NL/SI	82	35	30	23
	DK/HU	44	33	29	15
	SE/HE	54	47	34	8
	PT/PE	45	36	29	14
	PT/PI	55	39	31	15
UNITED STATES	WA/VO	52	41	29	28
	WA/SM	67	35	27	27
	MI/RE	52	34	27	23
	MI/LL	34	30	27	21
	ME/NO	32	25	16	5
	ME/WN	58	28	23	13
	ME/PQ	48	38	21	15
	MA/PT	62	33	21	20
	MA/CS	56	40	26	20
	NC/CC	43	40	20	14

Table S3: Confirmation of species identification using Sanger sequencing of partial 18S and Cox1 genes. Primers used for 18S were GS2-F and GS5-R [3] and for *Cox1* LCO1490 and HCO219 [4]. Only top hits are reported and matches *G. semen* and in one case *Chattonella marina var antiqua*. * Low quality sequence (low quality score leads to artificially low % identity. If gaps are ignored, then % identity is 99.4%; NA: Failed Sanger sequencing.

**18S ribosomal DNA**					***Cox1***				
**Sample**	**Species**	**Accession**	**% identity**	**E-value**	**Sample**	**Species**	**Accession**	**% identity**	**E-value**
BR27	*G. semen*	KP200894.1	99.6	0.0	BR27	*G. semen*	KP230751.1	99.7	0.0
CC36	*G. semen*	KP200894.1	99.2	0.0	CC36	*G. semen*	KP230753.1	90.1	0.0
CS02	*G. semen*	KP200894.1	99.5	0.0	CS02	*G. semen*	KP230751.1	98.5	0.0
GF14	*G. semen*	KP200894.1	99.1	0.0	GF14	*G. semen*	KP230751.1	99.7	0.0
HE42	*G. semen*	KP200894.1	99.8	0.0	HE42	*G. semen*	KP230751.1	99.7	0.0
HU32	*G. semen*	KP200894.1	99.5	0.0	HU32	*G. semen*	KP230751.1	99.7	0.0
KO51	*G. semen*	KP200894.1	99.2	0.0	KO51	*G. semen*	KP230751.1	99.4	0.0
LL30	*G. semen*	KP200894.1	99.8	0.0	LL30	*G. semen*	KP230751.1	87.3	0.0
NO20	*G. semen*	KP200894.1	99.6	0.0	NO20	*G. semen*	KP230751.1	98.3	0.0
NT33	*G. semen*	KP200894.1	99.5	0.0	NT33	*G. semen*	KP230751.1	99.7	0.0
PB44	*G. semen*	KP200894.1	99.5	0.0	PB44	*G. semen*	KP230751.1	99.7	0.0
PE30	*G. semen*	KP200894.1	99.6	0.0	PE30	*G. semen*	KP230751.1	99.7	0.0
PI25	*G. semen*	KP200894.1	99.6	0.0	PI25	*G. semen*	KP230751.1	99.7	0.0
PQ29	*G. semen*	KP200894.1	99.1	0.0	PQ29	*NA*	NA	NA	NA
PS12	*G. semen*	KP200894.1	96.139	0.0	PS12	*G. semen*	KP230751.1	99.681	0.0
PT27	*G. semen*	KP200894.1	98.910	0.0	PT27	*G. semen*	KP230751.1	99.683	0.0
RE08	*G. semen*	KP200894.1	99.510	0.0	RE08	*G. semen*	KP230751.1	98.574	0.0
RM25	*G. semen*	KP200894.1	99.634	0.0	RM25	*G. semen*	KP230753.1	94.620	0.0
RO49	*G. semen*	KP200894.1	99.384	0.0	RO49	*G. semen*	KP230751.1	99.683	0.0
SE25	*G. semen*	KP200894.1	99.018	0.0	SE25	*G. semen*	KP230751.1	99.684	0.0
SI24	NA	NA	NA	NA	SI24	*G. semen*	KP230751.1	99.521	0.0
SM27	*G. semen*	KP200894.1	99.626	0.0	SM27	*C. antiqua*	AF037990.1	84.088	0.0
VO34	*G. semen*	KP200894.1	99.751	0.0	VO34	*G. semen*	KP230751.1	99.525	0.0
VP19	*G. semen*	KP200894.1	99.755	0.0	VP19	*G. semen*	KP230751.1	99.522	0.0
WN14	*G. semen*	KP200894.1	94.585*	0.0	WN14	-	-	-	-

Table S4: RAD P1 adapter sequences with 48 different 8bp inline barcodes; top oligo nucleotide with 3’ phosphorothioate linkage between outermost and 2nd outermost nucleotide (∗) and bottom oligo nucleotide with 5’ phosphorylation (Phos).

P1 adapter top sequence:

5´-AATGATACGGCGACCACCGAGATCTACACTCTTTCCCTACACGACGCTCTTCCGATCTXXXXXXXXTGC∗A-3´

P1 adapter bottom sequence:

5´-Phos-XXXXXXXXAGATCGGAAGAGCGTCGTGTAGGGAAAGAGTGTAGATCTCGGTGGTCGCCGTATCATT-3´

P1 adapter	barcode	P1 adapter	barcode
1	CGATAGAA	25	CATGTGTT
2	CAACACAA	26	TGTCTAGT
3	AATGCCAA	27	GTACATGT
4	GCTAGATA	28	TCAGTTGT
5	GATGCTTA	29	TACGAGGT
6	TGGTAGTA	30	ATCATGGT
7	CTGTTGTA	31	TCACTACT
8	AGGTAAGA	32	GCGAGTAG
9	AGGATGGA	33	GGTTCGAG
10	TCTACGGA	34	ATGAACAG
11	ACACTCGA	35	CTCTGCAG
12	GTGTGCGA	36	ACCTCCAG
13	ACACAACA	37	GCATGTTG
14	CTCTCACA	38	CCACCTTG
15	GATTGTCA	39	GTAGTGTG
16	GGACCTCA	40	TCATTCTG
17	GTTCGGCA	41	CCTCTCTG
18	AACGGAAT	42	TACATAGG
19	TCGGCAAT	43	ATGGCAGG
20	TGCCTTAT	44	CACTCTGG
21	CAATGGAT	45	GGAAGACG
22	CGTGTCAT	46	AAGGATCG
23	ACTCGCAT	47	TACTACCG
24	GGAACATT	48	TTCATCCG

Table S5: RAD P2 adapter sequences with 6 different index barcodes; top oligo nucleotide with with 5’ phosphorylation (Phos) and bottom oligo nucleotide 3’ phosphorothioate linkage between outermost and 2nd outermost nucleotide (∗).

P2 adapter top sequence:
5’–Phos–GATCGGAAGAGCACACGTCTGAACTCCAGTCACXXXXXXXXATCAGAACAA–3’

P2 adapter bottom sequence:
5’–CAAGCAGAAGACGGCATACGAGATXXXXXXXXGTGACTGGAGTTCAGACGTGTGCTCTTCCGATC∗T–3’

Index barcode A: CGCTCATT

Index barcode B: GAGATTCC

Index barcode C: ATTCAGAA

Index barcode D: TAATGCGC

Index barcode E: CGGCTATG

Index barcode F: TCCGCGAA

Table S6: Population genetic metrics of *G. semen* lake populations in Europe and in the United States. Analysis based on a total number of 2542 variant sites from 360 individuals. Table content: population ID, number of individuals in each population, number of all sites (variant and fixed), percentage of polymorphic sites, number of private alleles, number of variant sites, nucleotide diversity π, observed heterozygosity H_o, expected heterozygosity H_e, inbreeding coefficient F_{is}.

Pop.	Indiv.	All sites	Priv. alleles	Var. sites	% Polym. loci	pi	H_o	H_e	F_{is}
EUR/CZ/KO	14	2,144,744	6	2385	0.010	0.023	0.018	0.022	0.012
EUR/CZ/RM	11	798,429	74	859	0.030	0.093	0.094	0.087	0.000
EUR/DE/GF	11	2,166,464	7	2412	0.005	0.009	0.010	0.008	-0.001
EUR/DE/RO	21	2,153,043	12	2393	0.011	0.022	0.018	0.022	0.013
EUR/DK/HU	15	2,157,826	10	2402	0.011	0.028	0.020	0.027	0.022
EUR/EE/PS	7	2,098,388	4	2326	0.008	0.023	0.018	0.021	0.012
EUR/EE/VP	9	2,051,043	8	2288	0.009	0.024	0.015	0.022	0.021
EUR/LT/NT	16	2,165,782	7	2417	0.009	0.018	0.013	0.017	0.015
EUR/LT/PB	25	2,108,223	76	2347	0.029	0.036	0.022	0.035	0.068
EUR/NL/BR	34	2,181,918	24	2433	0.022	0.039	0.027	0.038	0.039
EUR/NL/SI	23	2,191,006	21	2449	0.024	0.047	0.032	0.046	0.050
EUR/PL/SE	13	1,963,647	12	2184	0.010	0.022	0.015	0.020	0.018
EUR/PT/PE	14	2,068,078	7	2305	0.007	0.015	0.016	0.014	-0.002
EUR/PT/PI	15	2,056,651	9	2297	0.006	0.014	0.013	0.013	0.003
EUR/SE/HE	8	1,942,329	3	2177	0.009	0.028	0.017	0.025	0.024
USA/MA/CS	20	2,109,517	66	2344	0.021	0.050	0.030	0.048	0.055
USA/MA/PT	20	2,119,028	81	2357	0.023	0.053	0.036	0.051	0.049
USA/ME/NO	5	2,139,090	144	2381	0.016	0.051	0.037	0.045	0.033
USA/ME/PQ	15	2,077,430	132	2300	0.026	0.055	0.036	0.052	0.056
USA/ME/WN	13	2,037,030	79	2261	0.021	0.049	0.029	0.046	0.056
USA/MI/RE	23	2,066,059	121	2263	0.028	0.071	0.072	0.069	0.005
USA/WO/VO	28	2,173,309	157	2402	0.031	0.070	0.077	0.068	-0.012

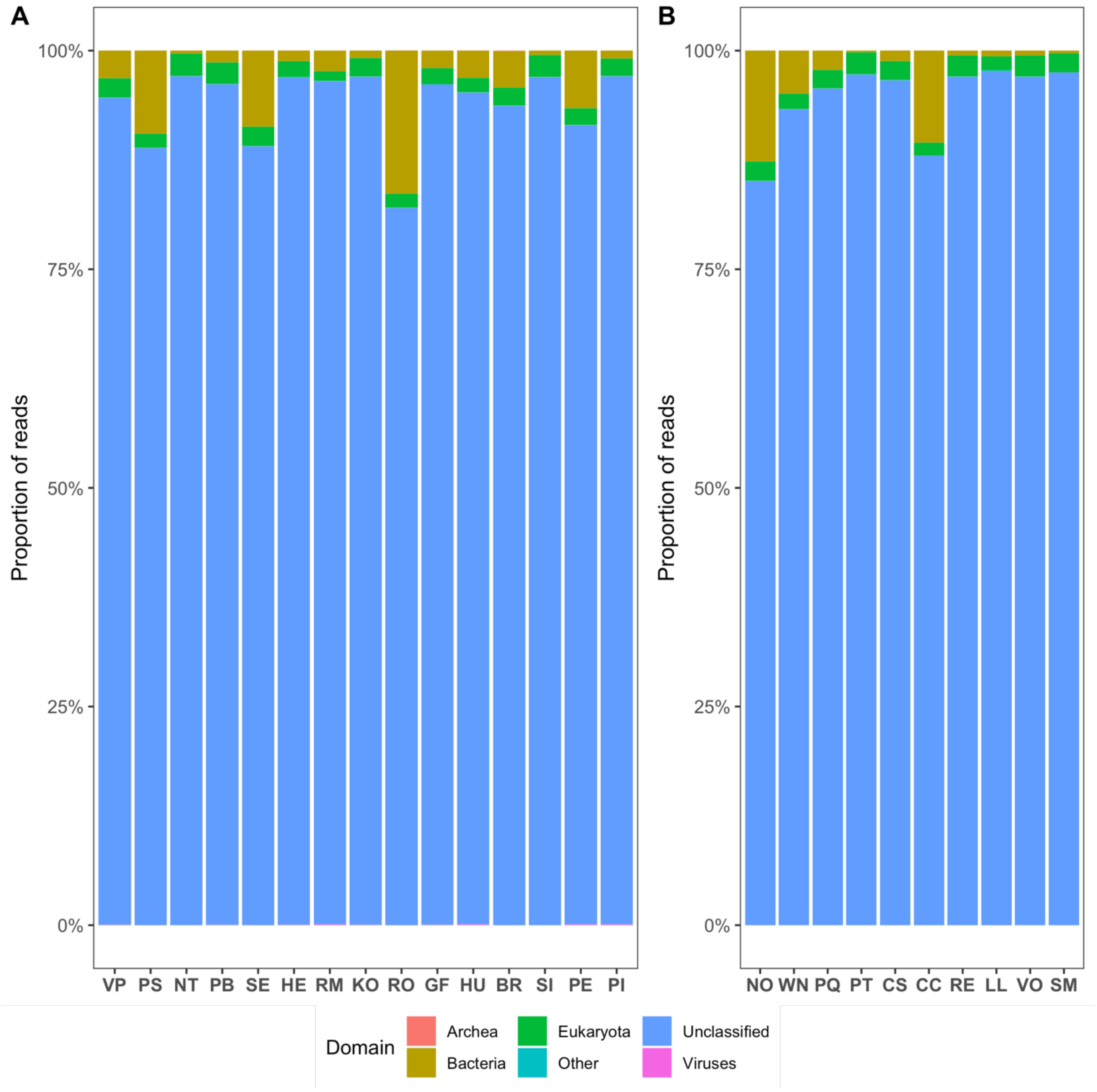

Figure S1: Results of Kraken taxonomic sequence classification per *Gonyostomum semen* sampling site in Europe (A) and in the United States (B). Reads were mainly classified as of human or bacterial origin, with very few reads attributed to archaea, viruses, or other taxa (< 0.01%). Kraken classification summary (domain level): bacteria: 3.58%; eukaryotes: 2.01%; viruses: 0.0272%, archaea: 0.00910%; other: 0.0495%; unclassified (including *Gonyostomum semen* reads): 94.32%

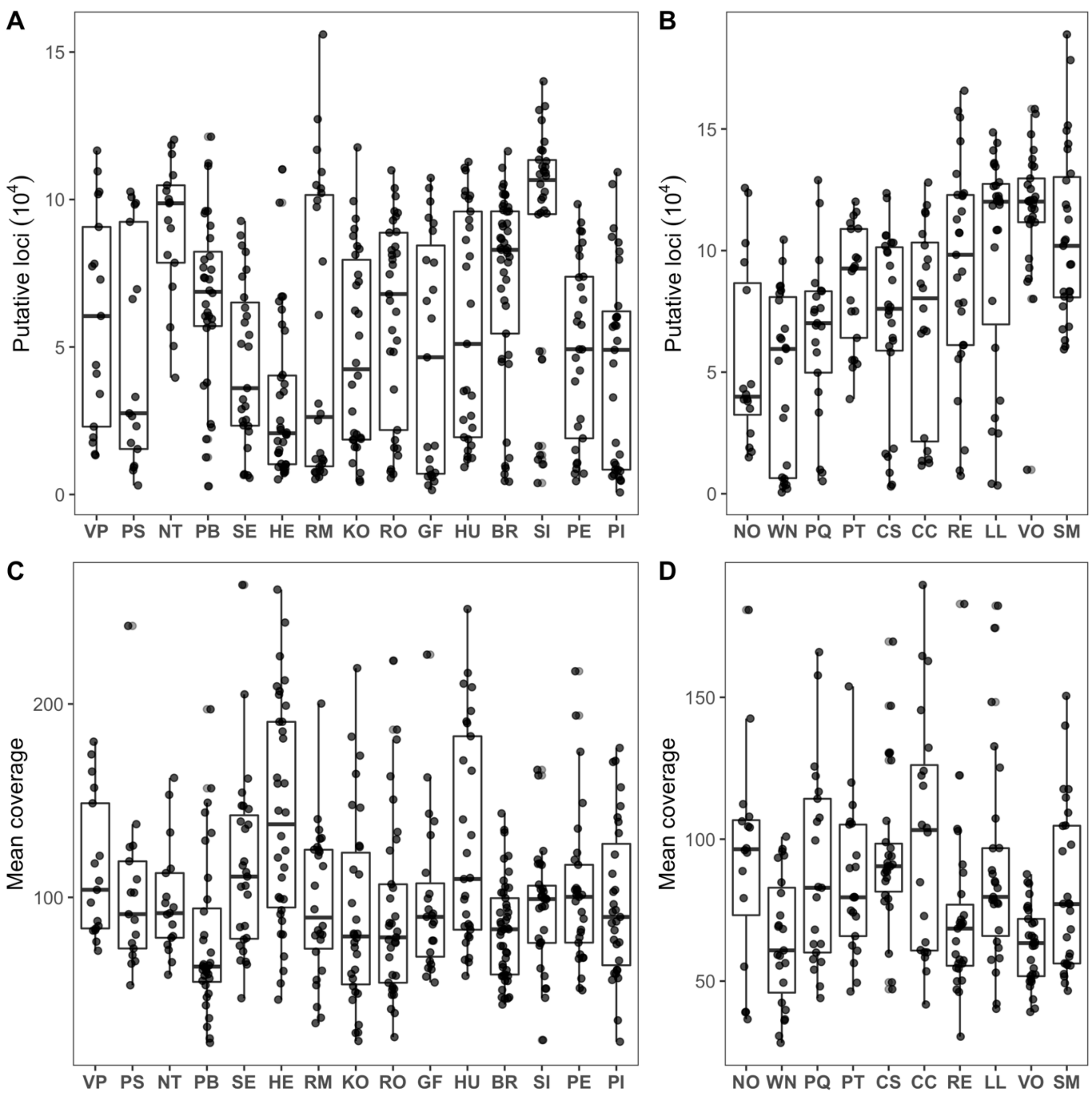

Figure S2: Number of stacks (putative loci) per sample (A: Europe, B: United States) and mean coverage (C: Europe, D: United States) in of G. semen lake populations in Europe and in the United Sates from Stacks ustacks analysis. Overall, the mean number of putative loci was 66,643 with a mean read coverage of 94.6x.

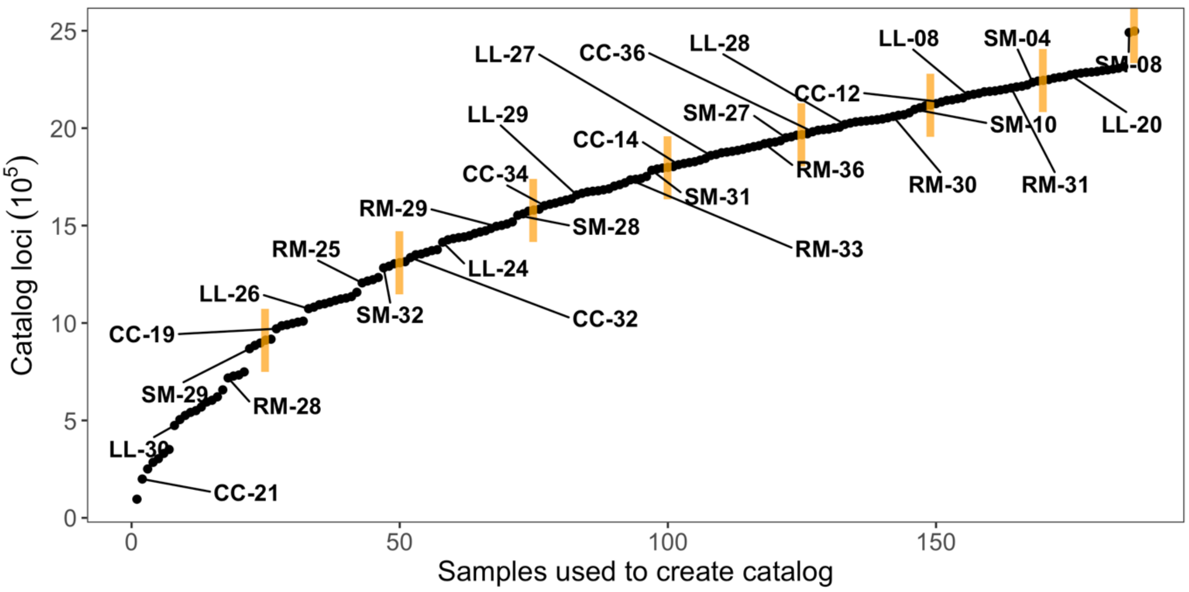

Figure S3: Size of the catalog of RADtags created in Stack cstacks relative to the number of *G. semen* samples from different lake populations in Europe and in the United States used to build the catalog. Orange lines indicate the different steps in catalog creation including 25, 50, 75, 100, 125, 149, 170 and 187 samples. Some labeled samples of lakes NC/CC, MI/LL, and WA/SM in the United States and lake CZ/RM in Europe thereby add a disproportionate number of new loci to the catalog.

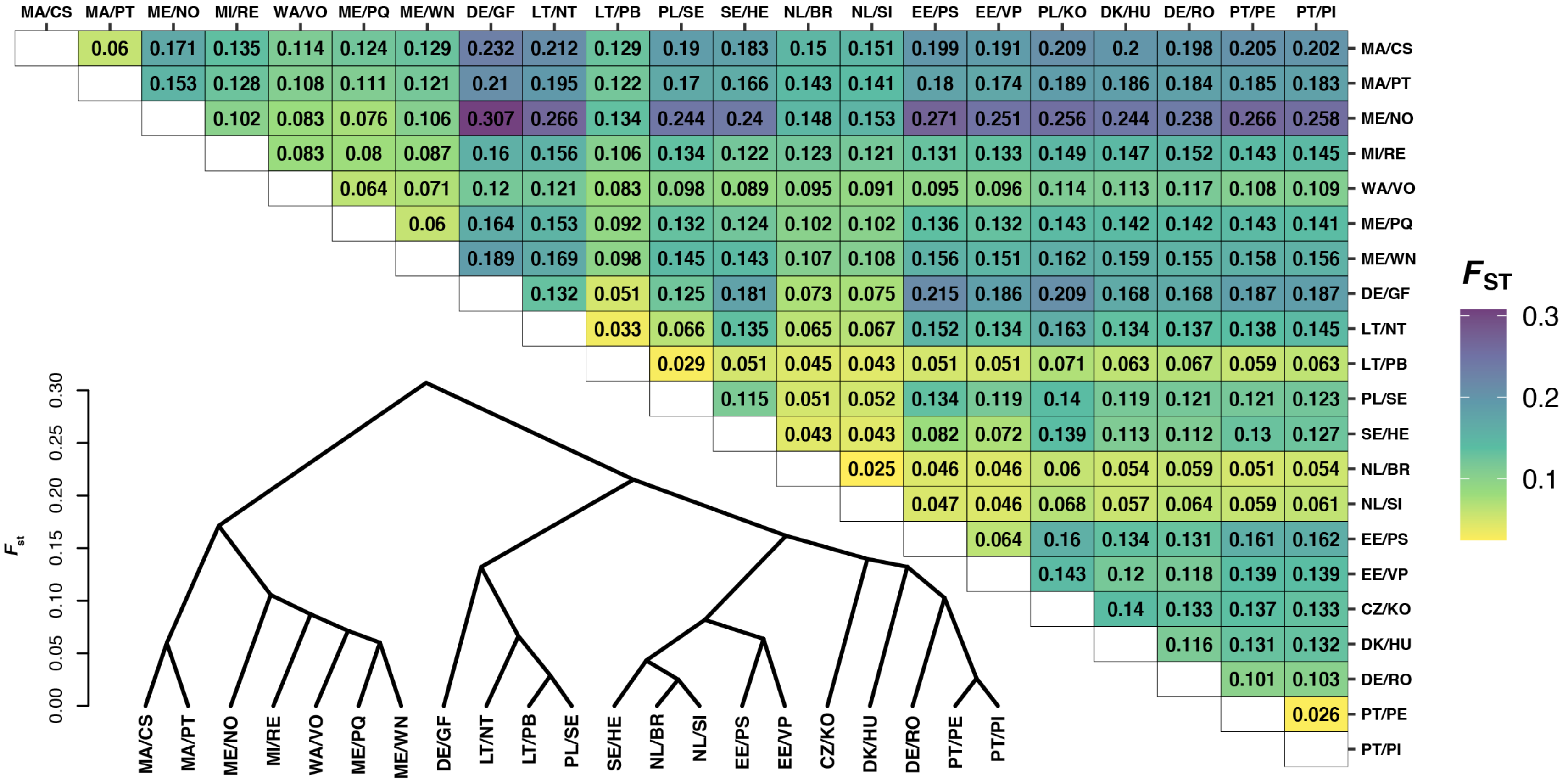

	MA/CS	MA/PT	ME/NO	MI/RE	WA/VO	ME/PQ	ME/WN	DE/GF	LT/NT	LT/PB	PL/SE	SE/HE	NL/BR	NL/SI	EE/PS	EE/VP	PL/KO	DK/HU	DE/RO	PT/PE	PT/PI
MA/CS		0.06	0.171	0.135	0.114	0.124	0.129	0.232	0.212	0.129	0.19	0.183	0.15	0.151	0.199	0.191	0.209	0.2	0.198	0.205	0.202
MA/PT			0.153	0.128	0.108	0.111	0.121	0.21	0.195	0.122	0.17	0.166	0.143	0.141	0.18	0.174	0.189	0.186	0.184	0.185	0.183
ME/NO				0.102	0.083	0.076	0.106	0.307	0.266	0.134	0.244	0.24	0.148	0.153	0.271	0.251	0.256	0.244	0.238	0.266	0.258
MI/RE					0.083	0.08	0.087	0.16	0.156	0.106	0.134	0.122	0.123	0.121	0.131	0.133	0.149	0.147	0.152	0.143	0.145
WA/VO						0.064	0.071	0.12	0.121	0.083	0.098	0.089	0.095	0.091	0.095	0.096	0.114	0.113	0.117	0.108	0.109
ME/PQ							0.06	0.164	0.153	0.092	0.132	0.124	0.102	0.102	0.136	0.132	0.143	0.142	0.142	0.143	0.141
ME/WN								0.189	0.169	0.098	0.145	0.143	0.107	0.108	0.156	0.151	0.162	0.159	0.155	0.158	0.156
DE/GF									0.132	0.051	0.125	0.181	0.073	0.075	0.215	0.186	0.209	0.168	0.168	0.187	0.187
LT/NT										0.033	0.066	0.135	0.065	0.067	0.152	0.134	0.163	0.134	0.137	0.138	0.145
LT/PB											0.029	0.051	0.045	0.043	0.051	0.051	0.071	0.063	0.067	0.059	0.063
PL/SE												0.115	0.051	0.052	0.134	0.119	0.14	0.119	0.121	0.121	0.123
SE/HE													0.043	0.043	0.082	0.072	0.139	0.113	0.112	0.13	0.127
NL/BR														0.025	0.046	0.046	0.06	0.054	0.059	0.051	0.054
NL/SI															0.047	0.046	0.068	0.057	0.064	0.059	0.061
EE/PS																0.064	0.16	0.134	0.131	0.161	0.162
EE/VP																	0.143	0.12	0.118	0.139	0.139
CZ/KO																		0.14	0.133	0.137	0.133
DK/HU																			0.116	0.131	0.132
DE/RO																				0.101	0.103
PT/PE																					0.026
PT/PI																					

Figure S4: Pairwise genetic distance (F_{ST} values) between lake populations in Europe and in the United States. Upper right: Heatmap table of F_{ST} values. Lower left: Hierarchical clustering tree that was used to arrange the order of population in the heatmap table. F_{ST} values between population CZ/RM and all other populations ranged from 0.24 to 0.4 and were omitted from the heatmap table. The FST values ranged from below 0.03 between lakes in close geographic proximity in Portugal, the Netherlands, Poland and Lithuania up to around 0.3 between lake DE/GF in Europe and ME/NO in the United States.

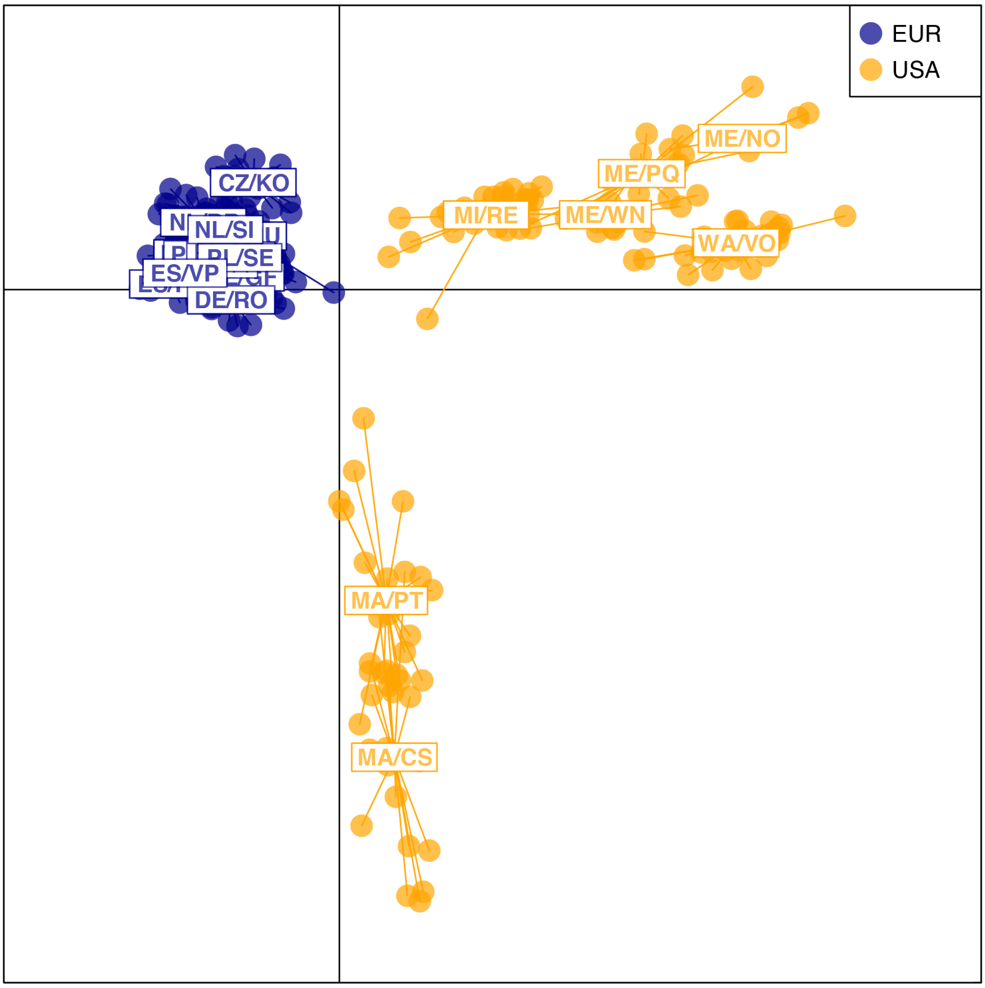

Figure S5: DAPC of *Gonyostomum semen* lake populations in Europe (CZ/RM excluded) and in the United States (NC/CC excluded). Populations are used as prior groupings and 14 PCs are retained (as evaluated through cross-validation and a-score). Points represent individuals and lines connect individuals within populations. The US populations were split into two separate clusters, one of which contained the two populations in Massachusetts (MA/CS and MA/PT) and the second cluster comprised all other populations (from Washington, Michigan, and Maine). All European populations formed one overlapping cluster.

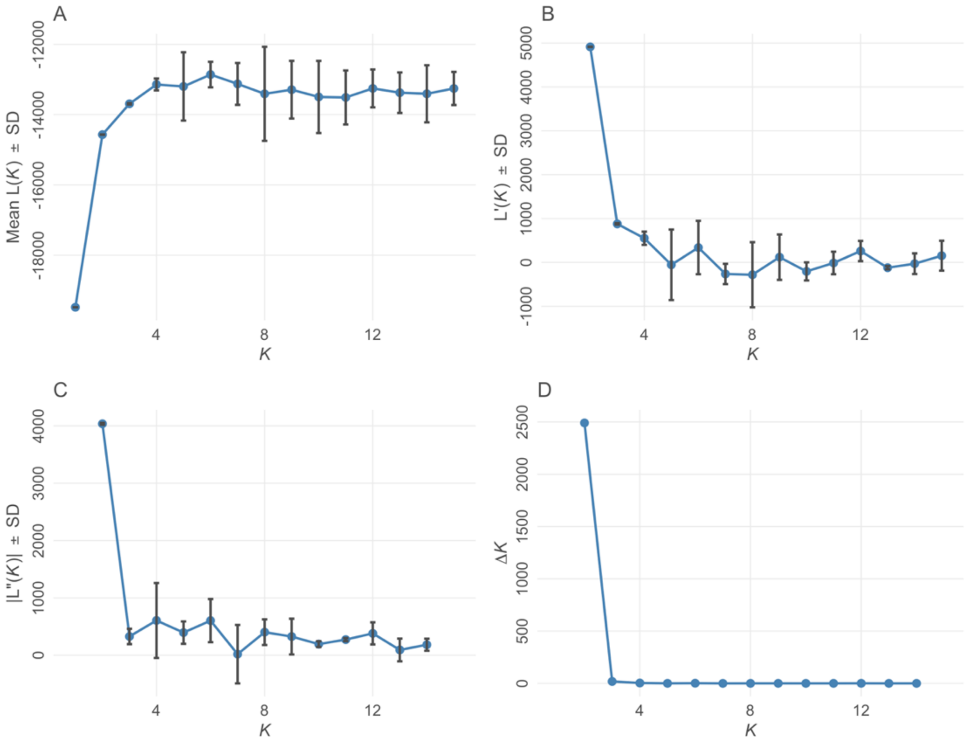

Figure S6: Results of the Evanno method using the R package pophelper v2.3.1 to determine the best number of clusters K in the Structure analysis of *G. semen* lake populations in Europe.

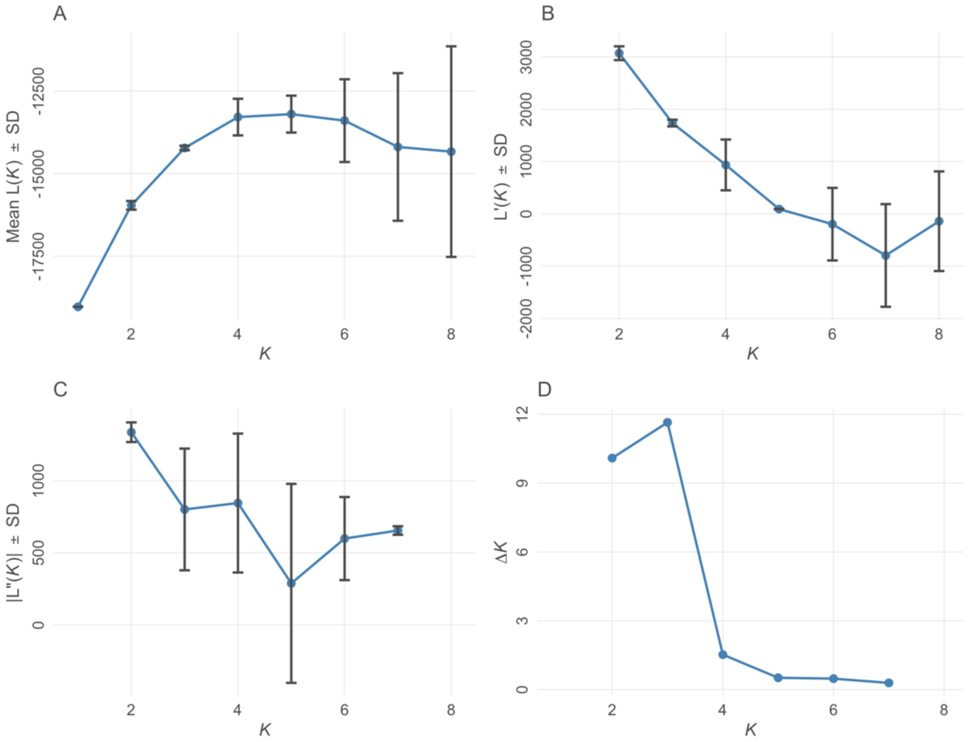

Figure S7: Results of the Evanno method using the R package pophelper v2.3.1 to determine the best number of clusters K in the Structure analysis of *G. semen* lake populations in the United States.

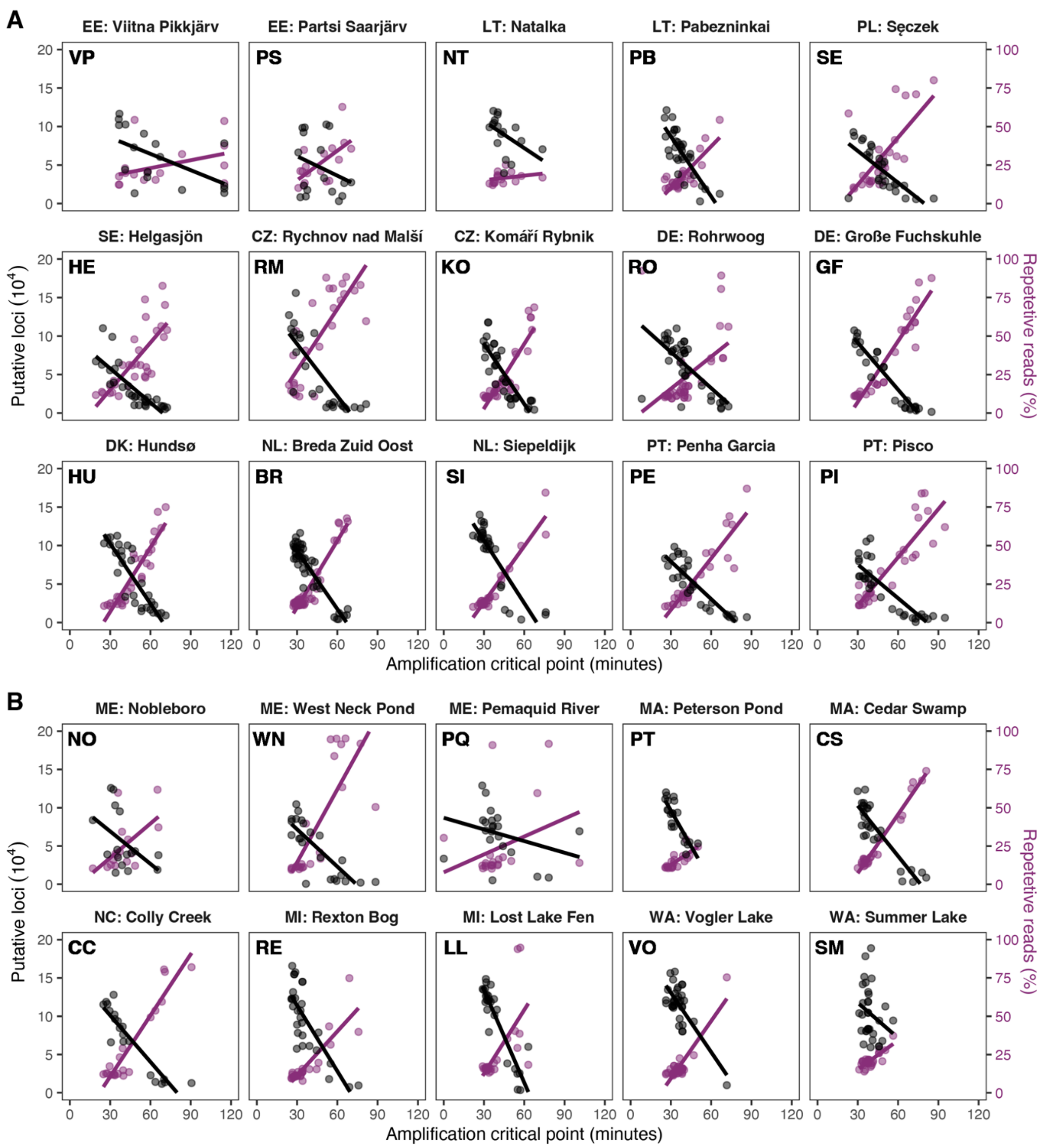

Figure S8: Recovery of putative loci (black) through RADseq from single amplified genomes of *Gonyostomum semen* relative to critical point estimates of amplification curves. The number of reads attributed to loci that were filtered out due to extreme coverage is shown in purple. (A) Samples from lakes in Europe and (B) the United States. European countries: Estonia (EE), Lithuania (LT), Poland (PL), Sweden (SE), Czech Republic (CZ), Germany (DE), Denmark (DK), Netherlands (NL), Portugal (PT); US states: Maine (ME), Massachusetts (MA), North Carolina (NC), Michigan (MI), Washington (WA).

References

1. Gollnisch R, Alling T, Stockenreiter M, Ahrén D, Grabowska M, Rengefors K. Calcium and pH interaction limits bloom formation and expansion of a nuisance microalga. Limnology and Oceanography. 2021;66:3523-34.

2. Buck U, Babenzien HD, Zwirnmann E. Extracellular peroxidase activity in an experimentally divided lake (Grosse Fuchskuhle, northern Germany). Aquat Microb Ecol. 2008;51:97-103.

3. Johansson KSL, Lührig K, Klaminder J, Rengefors K. Development of a quantitative PCR method to explore the historical occurrence of a nuisance microalga under expansion. Harmful Algae. 2016;56:67-76.

4. Folmer O, Black M, Hoeh W, Lutz R, Vrijenhoek R. DNA primers for amplification of mitochondrial cytochrome c oxidase subunit I from diverse metazoan invertebrates. Molecular Marine Biology and Biotechnology. 1994;3:294-9.
